# CaWRKY27 Negatively Regulates H_2_O_2_-Mediated Thermotolerance in Pepper (*Capsicum annuum*)

**DOI:** 10.3389/fpls.2018.01633

**Published:** 2018-11-19

**Authors:** Fengfeng Dang, Jinhui Lin, Baoping Xue, Yongping Chen, Deyi Guan, Yanfeng Wang, Shuilin He

**Affiliations:** ^1^National Education Minister, Key Laboratory of Plant Genetic Improvement and Comprehensive Utilization, Fujian Agriculture and Forestry University, Fuzhou, China; ^2^College of Crop Science, Fujian Agriculture and Forestry University, Fuzhou, China; ^3^Key Laboratory of Applied Genetics of Universities in Fujian Province, Fuzhou, China; ^4^College of Life Science, Yan’an University, Yan’an, China; ^5^College of Horticulture, Fujian Agriculture and Forestry University, Fuzhou, China

**Keywords:** *Capsicum annuum*, abiotic stress, thermotolerance, *CaWRKY27*, H_2_O_2_

## Abstract

Heat stress, an important and damaging abiotic stress, regulates numerous WRKY transcription factors, but their roles in heat stress responses remain largely unexplored. Here, we show that pepper (*Capsicum annuum*) CaWRKY27 negatively regulates basal thermotolerance mediated by H_2_O_2_ signaling. *CaWRKY27* expression increased during heat stress and persisted during recovery. *CaWRKY27* overexpression impaired basal thermotolerance in tobacco (*Nicotiana tabacum*) and *Arabidopsis thaliana*, *CaWRKY27*-overexpressing plants had a lower survival rate under heat stress, accompanied by decreased expression of multiple thermotolerance-associated genes. Accordingly, silencing of *CaWRKY27* increased basal thermotolerance in pepper plants. Exogenously applied H_2_O_2_ induced *CaWRKY27* expression, and *CaWRKY27* overexpression repressed the scavenging of H_2_O_2_ in *Arabidopsis*, indicating a positive feedback loop between H_2_O_2_ accumulation and *CaWRKY27* expression. Consistent with this, *CaWRKY27* expression was repressed under heat stress in the presence H_2_O_2_ scavengers and *CaWRKY27* silencing decreased H_2_O_2_ accumulation in pepper leaves. These changes may result from changes in levels of reactive oxygen species (ROS)-scavenging enzymes, since the heat stress-challenged *CaWRKY27*-silenced pepper plants had significantly higher expression of multiple genes encoding ROS-scavenging enzymes, such as *CaCAT1*, *CaAPX1*, *CaAPX2*, *CaCSD2*, and *CaSOD1*. Therefore, CaWRKY27 acts as a downstream negative regulator of H_2_O_2_-mediated heat stress responses, preventing inappropriate responses during heat stress and recovery.

## Introduction

High temperatures can damage plants, causing membrane injury, inactivating proteins, increasing production of reactive oxygen species (ROS), and damaging key metabolic functions (Quinn, 1988; Iba, 2002; Wahid, 2007). This abiotic stress leads to heavy crop losses, threatens food security, and is of increasing concern due to global climate change. Because of their sessile lifestyles, plants inevitably encounter heat stress (HS) and have evolved defense mechanisms to help them cope with this stress. These mechanisms include perception of HS, initiation and transduction of defense signals, and massive transcriptional reprogramming by various transcription factors (TFs). This transcriptional reprogramming leads to synthesis of heat shock proteins (HSPs) that ameliorate the protein misfolding and aggregation issues caused by HS (Queitsch et al., 2000). A variety of molecules are involved in signaling HS, including Ca^2+^ (Jia et al., 2014), phytohormones such as salicylic acid (Clarke et al., 2004), jasmonic acid (Clarke et al., 2009), ethylene (Larkindale and Huang, 2004), and abscisic acid (Larkindale and Huang, 2004), and ROS such as H_2_O_2_ (Zang et al., 2017; Salvi et al., 2018) and NO (Wang et al., 2014).

Transcription factors play crucial roles in the HS response by responding to upstream defense signals and transcriptionally modulating the expression of thermotolerance-associated genes (Fragkostefanakis et al., 2015; Ohama et al., 2017). For example, HSFA6b plays a role in the HS response by controlling the expression of HSPs in response to abscisic acid signaling (Huang et al., 2016). SPL1 and SPL12 contribute to heat-triggered transcriptional reprogramming (Chao et al., 2017). Upon interacting with phosphatases, the RCF2 and NAC019 transcriptionally regulate the expression of *HSFA1b*, *HSFA6b*, *HSFA7a*, and *HSFC1*, which encode TFs that transcriptionally regulate the expression of *HSPs* (Guan et al., 2014). These results demonstrate the important, complex role that TFs play in regulating thermotolerance, however, the roles of TFs in the plant HS response, and how they are connected to the upstream signaling components, has yet to be elucidated.

Reactive oxygen species are produced by NADPH oxidases (termed respiratory burst oxidase homologs, RBOHs) in the apoplast, and by oxidases and peroxidases in the chloroplast, mitochondria, peroxisome, and possibly other cellular compartments (Suzuki et al., 2011; Mignolet-Spruyt et al., 2016). They can also be scavenged by ROS-detoxifying enzymatic proteins such as superoxide dismutase (SOD), ascorbate peroxidase (APX), catalase (CAT), glutathione peroxidase (GPX), and peroxiredoxin (PRX), or by antioxidants such as ascorbic acid and glutathione (Davletova et al., 2005). The synthesis and scavenging of ROS is balanced in healthy plants. However, this balance is frequently upset when plants are challenged by various stressors, resulting in a ROS burst that causes oxidative stress, which includes oxidative damage to membranes, proteins, RNA, and DNA molecules, and potentially the oxidative destruction of the cell (Mittler, 2002). ROS also serve as signal transduction molecules (Choudhury et al., 2017; Salvi et al., 2018). Some TFs such as HsfA1a directly sense H_2_O_2_ and modulate the transcription of genes involved in the plant HS response. However, our knowledge of the connection between H_2_O_2_ and the various TFs that regulate thermotolerance is very limited.

WRKY TFs are one of the largest TF families in plants and have been implicated as positive or negative regulators of growth, development, and responses to the environment. WRKYs are classified based the presence of one or two highly conserved WRKY domains as well as their specific binding to conserved cognate W-boxes [TTGAC (C/T)]. Significant differences in *WRKY* gene expression under HS were observed for 30 of the 36 tested *WRKYs* in radish (Karanja et al., 2017) and 17 of the 22 tested *WRKYs* enhanced their expression in potato (Zhang et al., 2017), indicating that multiple WRKY TFs participate in the regulation of the plant HS response. Multiple WRKY TFs function in thermotolerance by modulating the expression of heat-inducible and oxidative stress-responsive genes *Hsp*, *Hsf*, *PR1*, and *MBF1c* (Li et al., 2010, 2011). These characterized WRKY TFs include *AtWRKY25* (Li et al., 2009), *AtWRKY26*, *AtWRKY33* (Li et al., 2011), *AtWRKY39* (Li et al., 2010), *CaWRKY6* (Cai et al., 2015), *CaWRKY40* (Dang et al., 2013), *TaWRKY1*, *TaWRKY33* (He et al., 2016), and *OsWRKY11* (Wu et al., 2009), which regulate the plant HS response. However, since only a subsets of WRKY TFs that are involved in regulating the HS response have been characterized, the roles of the remaining WRKY TFs remain to be elucidated.

Pepper (*Capsicum annuum*) is an agriculturally important crop from the *Solanaceae*. HS not only adversely affects its growth and development, but also increases its susceptibility to disease when grown under high humidity conditions. A better understanding of the mechanism of thermotolerance in pepper has potential applications for the genetic improvement of its heat tolerance. Previously, we showed that *CaWRKY6* (Cai et al., 2015), and *CaWRKY40* (Dang et al., 2013) act as positive regulators of the HS response in pepper and *Ralstonia solanacearum* infection (*RSI*), and that *CaWRKY6* regulates the expression of *CaWRKY40* in these responses (Cai et al., 2015). *CaWRKY27* is induced by *RSI* as well as exogenously applied salicylic acid, methyl jasmonate, or ethylene, and its overexpression in tobacco conferred resistance to *RSI*. Virus-induced gene silencing (VIGS) of *CaWRKY27* in pepper attenuated its resistance to *RSI* (Dang et al., 2014). In this study, the results from gain-of-function and loss-of-function analyses indicate that *CaWRKY27* also acts as a crucial negative regulator of basal thermotolerance in pepper via the H_2_O_2_-mediated signaling pathway, and plays a significant role in coordinating disease resistance.

## Materials and Methods

### Plant Materials and Growth Conditions

Seeds from *Capsicum annuum* #8 (provided by the pepper breeding group of Fujian Agriculture and Forestry University) and tobacco (*Nicotiana tabacum*) cultivar K326 (provided by Tobacco Institute of Fujian Tobacco Company) were soaked in water at 25 ± 2°C°C overnight, and then were sown into a steam-sterilized soil mix (peat moss and vermiculite, 1/1, v/v) in plastic pots. Plants were grown in a growth room that was maintained at 25 ± 2°C with a light intensity of ∼100 μmol photons m^−2^s^−1^ and a relative humidity of 70% under a 16-h-light/8-h-dark cycle.

Wild-type (*Col-0*) and transgenic *Arabidopsis* seeds were vernalized for 3 days in the dark at 4°C and transferred onto ½-strength MS and 0.8% agar plates that were incubated in a growth chamber (22 ± 2°C, ∼100 μmol photons m^−2^ s^−1^, relative humidity 85%, and 16-h light/8-h dark cycle).

### Construction of Transgenic Plants

To construct the *35S* promoter-driven *CaWRKY27*-overexpression construct, the coding region of *CaWRKY27* was PCR amplified using the primers *CaWRKY27-CSDF* and *CaWRKY27-CDSR*, and then cloned into the pK7WG2 vector using the gateway system (Invitrogen) according to the manufacturer’s instructions. To construct the *CaWRKY27* promoter-*GUS* fusion, fragments upstream of *CaWRKY27* were amplified from pepper genomic DNA using PCR and were cloned into the pMDC163 vector (Invitrogen). Each construct was introduced separately into *Agrobacterium tumefaciens* GV3101, which was then used to transform *Arabidopsis* with the floral dip method. Transgenic lines were selected by germinating seeds on 1/2 MS medium containing kanamycin (50 mg/L) or hygromycin (50 mg/L) as required, then selfed, and only lines segregating the transgene in a 3:1 ratio were selected for further analysis. The T_3_ seeds from *CaWRKY27-OE4* and *CaWRKY27-OE9* tobacco K326 plants were obtained as described previously (Dang et al., 2013), and then used for phenotypic scoring under HS.

### Treatments and Growth Analysis

To investigate *CaWRKY27* transcript levels in pepper plants, six-leaf stage pepper plants were treated at 42°C as described previously (Guo et al., 2007; Dang et al., 2013), and pepper leaves were harvested at 0, 0.5, 1, 3, and 6 h, and at 2, 4, 8, and 12 h after recovery at 25°C. For the exogenous H_2_O_2_ application, six-leaf stage pepper plants were sprayed with 20 mM H_2_O_2_ and plants were collected 0, 1, 3, 6, 12, 24, 36, and 48 h later. To analyze *pCaWRKY27::GUS* transgenic lines, 7-day-old *Arabidopsis* seedlings were treated at 37°C for 1 h, H_2_O_2_ (10 mM for 2 h). For phenotypic analysis, the *CaWRKY27*-silenced pepper, *Arabidopsis*, or tobacco plants were treated with HS at various times and analyzed (detailed methods are described in the figure legends). For qRT-PCR analysis, approximately 50-day-old *CaWRKY27*-silenced pepper plants, approximately 1-month-old *Arabidopsis* plants, and 45-day-old tobacco plants were subjected to HS and were harvested at the indicated time points according to the method described in the figure legends. To assay the effect of oxygen species scavengers on H_2_O_2_ production during pepper response to HS, the detached pepper leaves were employed and were tiled on 1/2 MS medium with or without 10 mM ascorbic acid, 100 μM DPI and 100 μM quinacrine (Chen et al., 2013; Mellidou et al., 2017). For HS treatment, the treated pepper leaves were put to temperature of 42, 38, 35, or 33°C, it was found that temperature of 42, 38, and 35°C resulted in rapidly leaves death, difficulty in H_2_O_2_ detection, and quick RNA degradation. So the treatment under 33°C for 3 h and recover for 1/2 h was finally employed.

### Histochemical Staining

H_2_O_2_ accumulation was detected with DAB staining. Pepper or *Arabidopsis* leaves were soaked in 1 mg⋅mL^−1^ diaminobenzidine (DAB, Sigma) for 15 h, and were cleared by boiling in a 1:1:3 mixture of lactic acid:glycerol:absolute ethanol (V:V:V) followed by destaining overnight in absolute ethanol as described previously (Dang et al., 2013). Representative phenotypes were photographed with a light microscope (Leica, Germany). To detect GUS expression, the samples were immersed into GUS staining solution [1 mg⋅mL^−1^ X-Gluc, 1 mM K_3_Fe(CN)_6_, 1 mM K_4_Fe(CN)_6_, 50 mM sodium phosphate buffer pH 7.0, 10 mM Na_2_EDTA, 0.1% Triton X-100] and incubated overnight at 37°C. The chlorophyll was then removed with several washes with 75% ethanol and phenotypes were observed and documented with a stereoscope (Leica, Germany).

### Electrolyte Leakage Measurements

Electrolyte leakage assays in *Arabidopsis* (Clarke et al., 2004) and pepper (Kim et al., 2010) were performed as described previously. Briefly, leaf disks 4 cm in diameter were washed in sterile double-distilled water for 30 min with slight agitation for 2 h at 25°C, and electrolyte leakage was detected using a conductivity meter (METTLER TOLEDO, Switzerland).

### Virus-Induced Gene Silencing

*Tobacco rattle virus* (TRV) based VIGS was performed to generate *CaWRKY27*-silenced pepper plants (*PYL-279-wrky27, PYL-279-wrky27*-*3′utr*). Fragments of the *CaWRKY27* coding sequence or the *CaWRKY27* 3′ untranslated region (UTR) were cloned from pepper cDNA and inserted into the *PYL-279* vector using gateway cloning (Invitrogen) as described previously (Dang et al., 2013). Fully expanded cotyledons from ∼16-day-old pepper seedlings were co-infiltrated with *A. tumefaciens* strain GV3101 carrying *PYL-192* as well as *PYL-279-pds* (Dang et al., 2013), *PYL-279-wrky27*, or *PYL-279-wrky27*-*3′utr*. About 20 days later, a photobleaching phenotype was observed due to phytoene desaturase (PDS) silencing in the positive control pepper plants (*PYL-279-pds*), and the transcript levels of *CaWRKY27* were measured in *PYL-279-wrky27* and *PYL-279-wrky27*-*3′utr* pepper plants by qRT-PCR after exposure to HS.

### Gene Expression Analysis

Total RNA was extracted from *Arabidopsis*, pepper, or tobacco plants by using the TaKaRa MiniBEST Universal RNA Extraction Kit (TaKaRa, Japan). RNA (1 μg) was used to synthesize cDNA with the TaKaRa PrimeScript RT-PCR Kit (TaKaRa, Japan) according to the manufacturer’s instructions. Transcript levels were measured with a CFX96 real-time PCR instrument (Bio-Rad, United States), the SYBR Premix Ex Taq II reagent (TaKaRa Perfect Real Time), and specific primers (Supplementary Table S1). *Arabidopsis* *UBIQUITIN10* (*AtUBQ10*), tobacco *Elongation factor 1 alpha* (*NtEF1α*), and pepper *Actin1* (*CaActin1*) were used for normalization.

### Statistical Analysis

All experiments were performed using three biological triplicates. All the data are expressed as the mean ± SE. One-way analysis of variance (ANOVA) was used on the data sets and tested for significant (*P* < 0.05 and *P* < 0.01) treatment differences using Student–Newman–Keuls test.

## Results

### *CaWRKY27* Expression Was Induced in Pepper Plants During Heat Stress and During the Recovery From Heat Stress

**FIGURE 1:**
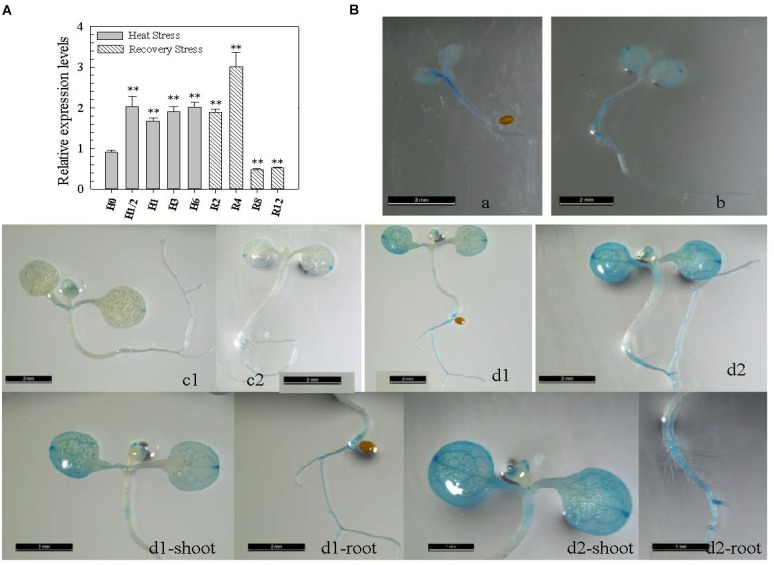
*CaWRKY27* was transcriptionally induced by heat treatment in pepper plants. **(A)** *CaWRKY27* expression in pepper leaves was determined with qRT-PCR at the indicated time points during or after heat treatment. Relative transcript levels of *CaWRKY27* in heat-treated pepper plants were compared to the control, which was set to a relative expression level of ‘1.’ Data represent the mean ± SE of three biological replicates, and asterisks indicate significant difference compared to control plants (*SNK*-test, ^∗^*P* < 0.05 or *^∗∗^P* < 0.01). H, heat; R, recovery. **(B)** GUS expression in transgenic *Arabidopsis* plants carrying the *pCaWRKY27::GUS* construct. Figures a, b, c1, and c2, show a seedling of 3 (a), 5 (b) and 7 (c1 and c2) days after germination (DAG). In figures d1 and d2 transgenic *pCaWRKY27::GUS* seedlings were heat treated. In figures d1-shoot, d1-root, d2-shoot, and d2-root the shoot or root was magnified from the corresponding d1 or d2 figures, respectively. These seedlings or plants were grown on ½ MS media under 16 h light/8 h dark conditions.

The presence of four HS elements (*HSEs*) in the *CaWRKY27* promoter region implies that it may be involved in the HS response (Supplementary Figure S1A); however, a role for *CaWRKY27* in pepper thermotolerance had not been reported. To test this speculation, we measured the transcript level of *CaWRKY27* in pepper leaves by qRT-PCR at different time points during or after treatment with HS. We found that the transcript abundance of *CaWRKY27* increased after 0.5 to 6 h at 42°C and that this increase persisted for an additional 4 h of recovery at room temperature (25°C). The maximum transcript abundance increase was approximately 3.0-fold compared to control plants and was observed after 4 h of recovery at room temperature (Figure 1A).

To confirm the increase in *CaWRKY27* expression upon exposure to HS, we examined *CaWRKY27* expression using a *promoter-GUS* fusion. To this end, we produced transgenic *A. thaliana* lines carrying the 2-kb region genomic region upstream of the *CaWRKY27* translational start codon fused to a *GUS* reporter gene. Ten independent homozygous single-copy *CaWRKY27 promoter-GUS* fusion lines were examined, and representative consensus expression patterns were described (Figure 1B). GUS expression in leaves and roots was extremely low in 7-day-old transgenic *Arabidopsis* seedlings that were not challenged by stress, and enhanced when plants were challenged with HS at 37°C for 1 h. Together, these results suggest that *CaWRKY27* might play a role in pepper thermotolerance. The high consistency between the results from qRT-PCR and *GUS* expression experiments indicate that the induction of *CaWRKY27* by HS.

### Silencing of *CaWRKY27* Enhanced the Tolerance of Pepper Plants to Heat Stress

The induction of *CaWRKY27* expression by HS in pepper plants suggests that *CaWRK27* may be involved in the HS response. To test this, we performed a knockdown experiment in which *CaWRKY27* gene expression was silenced by VIGS in pepper seedlings, followed by analysis of the physiological and molecular responses of the *CaWRKY27*-silenced pepper plants to HS. We used two constructs that targeted different regions of the *CaWRKY27* transcript, *PYL-279-wrky27*, which targets the open reading frame, and *PYL-279-wrky27-3′utr*, which targets the 3′ UTR. The efficiency of *CaWRKY27* silencing by VIGS was confirmed by qRT-PCR, which showed that the *CaWRK27* transcript level decreased by approximately 3.51-fold and 3.32-fold in the pepper plants inoculated with *PYL-279-wrky27* and *PYL-279-wrky27-3′utr*, respectively, compared to control plants inoculated with empty vector (*PYL-279*) under normal conditions and by or 3.56-fold and or 3.61-fold under HS (Supplementary Figure S1B).

**FIGURE 2:**
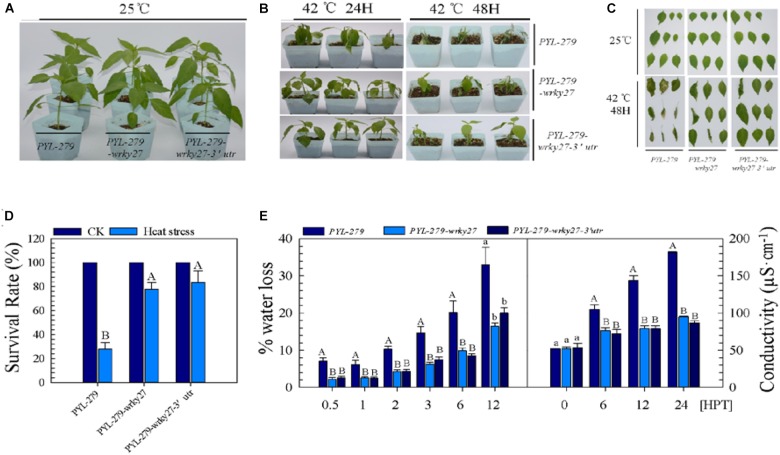
*CaWRKY27* silencing enhanced basal thermotolerance in pepper plants. **(A)** *PYL-279*, *PYL-279-wrky27*, and *PYL-279-wrky27-3*’*utr* pepper plants that were grown in pots for 6 weeks under normal conditions. **(B)** Phenotypes of *PYL-279*, *PYL-279-wrky27*, and *PYL-279-wrky27-3*’*utr* pepper plants after 3 days recovery following heat treatment at 42°C for 24 or 48 h. **(C)** Digital photographs of isolated leaves from the corresponding plants. **(D)** Survival rate were analyzed in *PYL-279*, *PYL-279-wrky27*, and *PYL-279-wrky27-3*’*utr* plant after 3 days recovery following heat treatment at 42°C for 48 h. Data represent the mean ± SE (*n* = 3), each replicate consists of six plants. **(E)** Percentage of water loss and ion leakage (conductivity) were detected in *PYL-279*, *PYL-279-wrky27*, and *PYL-279-wrky27-3*’*utr* leaves. Data represent the mean ± SE of three biological replicates. Different letters indicate significant differences compared to *PYL-279* plant leaves (*SNK*-test, lowercase letters indicate *P* < 0.05, and uppercase letters indicate *P* < 0.01).

We further examined the effect of *CaWRKY27* silencing on thermotolerance in pepper by investigating phenotypic changes in *PYL-279*, *PYL-279-wrky27*, and *PYL-279-wrky27-3′utr* plants in response to HS. No phenotypic differences were observed under normal condition (Figure 2A). However, when plants were challenged with HS (42°C) for 24 h and then returned to room temperature (25°C) to recover for 3 days, *PYL-279* plants exhibited a moderate wilted phenotype, while only slightly wilted phenotypes were observed in *PYL-279-wrky27* and *PYL-279-wrky27-3′utr* plants (Figure 2B). When the plants were challenged with HS (42°C) for 48 h and then returned to room temperature to recover for 3 days, *PYL-279* plants had a lower survival rate (27%) compared with *PYL-279-wrky27* (77%) and *PYL-279-wrky27-3′utr* (83%) plants (Figures 2B–D). Additionally, we found that the fresh weight loss of leaves due to HS-induced water loss was lower in *PYL-279-wrky27* and *PYL-279-wrky27-3′utr* plants than that in *PYL-279* plants. This was accompanied by significantly less electrolyte leakage in the leaves of *PYL-279-wrky27* and *PYL-279-wrky27-3′utr* plants than in the leaves of *PYL-279* plants when challenged with HS (Figure 2E). Together, these data suggest that *CaWRKY27* silencing enhanced the basal thermotolerance of pepper plants and that *CaWRKY27* might act as a negative regulator of basal thermotolerance in pepper.

### Overexpression of *CaWRKY27* Reduced Thermotolerance in Tobacco and *Arabidopsis*

**FIGURE 3:**
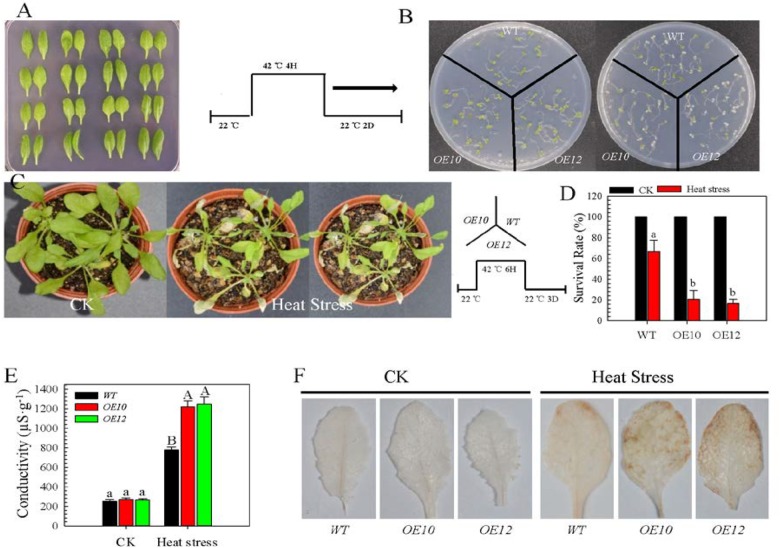
Overexpression of *CaWRKY27* reduced basal thermotolerance in transgenic *Arabidopsis* plants. **(A)** Leaves of *CaWRKY27-*overexpressing *Arabidopsis* and WT plants that were grown in pots for 5 weeks under normal conditions. **(B)** Phenotypes of *CaWRKY27-OE10*, *OE12*, and WT seedling that were treated at 37°C for 4 h and then returned to 22°C for 2 days of recovery. **(C)** Phenotypes of 35-day-old *CaWRKY27-OE10*, *OE12*, and WT plants that were treated at 37°C for 6 h and then returned to 22°C for 3 days of recovery. **(D)** Survival rate were analyzed in *CaWRKY27-OE10*, *OE12*, and WT plant after 3 days recovery following heat treatment at 37°C for 6 h. Data represent the mean ± SE (*n* = 3), each replicate consists of eight plants. **(E)** Electrolyte leakage in 28-day-old heat-treated seedlings was monitored by measuring conductivity. **(F)** H_2_O_2_ accumulation in leaves from heat-treated (37°C) WT, *CaWRKY27-OE10*, and *OE12* plants was measured by DAB staining. Data represent the mean ± SE of three biological replicates. Different letters indicate significant differences compared to WT (*SNK*-test, lowercase letters indicate *P* < 0.05, and uppercase letters indicate *P* < 0.01).

To confirm a role of *CaWRKY27* in thermotolerance, we generated transgenic *Arabidopsi*s that overexpression of *CaWRKY27* driven by the *CaMV 35S* promoter and obtained 14 homozygous T_4_ lines. Semi-quantitative PCR shows that the transcript levels of *CaWRKY27* were similar among all 14 T_4_ lines (Supplementary Figure S1C), although we did not observe any morphological differences between the T_4_ *CaWRKY27-OE* lines and wild-type (WT) plants (Figure 3A). Two independent T_4_ homozygous single-copy lines (*CaWRKY27-OE10* and *OE12*) that had moderate levels of *CaWRKY27* expression were used in further analyses. When *CaWRKY27-OE10* and *OE12* seedlings were grown on ½-strength Murashige and Skoog (MS) medium for 5 days, there was no significant morphological difference compared with the WT under normal conditions. However, upon HS (37°C) for 4 h followed by a 2 days recovery at 22°C, *CaWRKY27-OE10* and *OE12* displayed a decrease in thermotolerance compared to the WT (Figure 3B).

To directly assess the effect of *CaWRKY27* overexpression on HS tolerance, we subjected 3-week-old *CaWRKY27-OE10*, *OE12* and WT plants to 37°C for 6 h followed by a 3 days recovery at 22°C in a growth chamber. We observed that the WT plants grew much better under this condition (Figure 3C) and had higher survival rates (67%) compared to the *CaWRKY27-OE10* (20%) and *OE12* (16%) lines (Figure 3D). Accordingly, remarkably high electrolyte leakages were detected in *CaWRKY27-OE10* and *OE12* plants compared to the WT upon HS (Figure 3E). In addition, more H_2_O_2_ accumulation was detected in *CaWRKY27-OE10* and *OE12* plants than in WT plants under HS (Figure 3F).

**FIGURE 4:**
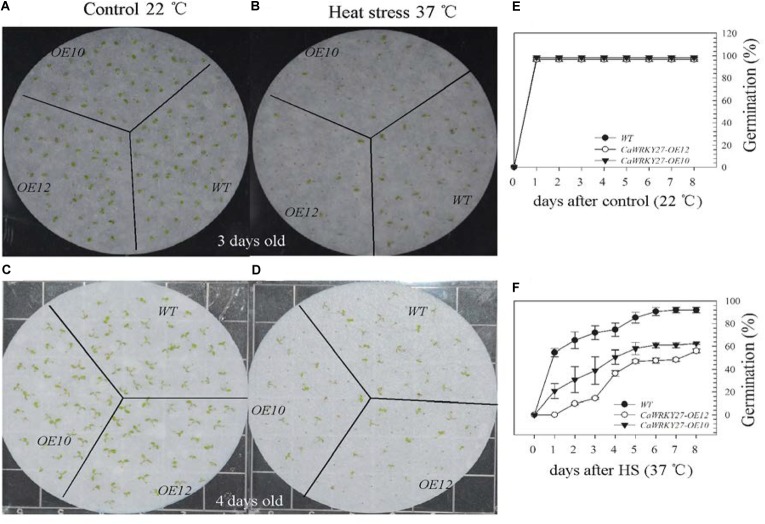
Responses of WT and *CaWRKY27*-overexpressing *Arabidopsis* lines to heat treatment during seed germination. **(A–D)** Illustration of representative seeds or seedlings at 3 or 4 days after heat treatment. **(E,F)** The percent of radicle emergence was recorded daily until no further radicles emerged in seeds of WT, *CaWRKY27-OE10*, and *OE12* plants. Seeds were treated at 37°C or at 22°C for the control. Data represent the mean ± SE (*n* = 3), each replicate consists of 30 seeds.

We also examined the seed germination of WT, *CaWRKY27-OE10*, and *OE12* under HS (37°C) after vernalization for 3 days at 4°C, and scored the percentages of radicle emergence daily until no further germination was observed. Two days after HS treatment, lower seed germination ratios were observed for *CaWRKY27-OE10* (38%) and *OE12* (14%) compared to the WT (72%), while 6 days after HS treatment, the seed germination ratios were 61% and 47% for *CaWRKY27-OE10* and *OE12*, respectively, and 90% for WT plants (Figures 4B,D,F). By contrast, no significant differences in seed germination were observed among WT, *CaWRKY27-OE10*, and *OE12* lines at room temperature (Figures 4A,C,E).

In parallel, we generated nine *CaWRKY27*-overexpressing T_2_ transgenic tobacco lines and assessed *CaWRKY27* transcript levels with qRT-PCR (Supplementary Figure S2A). Two independent lines (*OE4* and *OE9*) with high *CaWRKY27* expression levels were selected and used to generate T_3_ lines that were then used in phenotypic analyses. We first examined the survival rate of 15-day-old *CaWRKY27-OE4*, *OE9*, and WT seedlings that had been treated at 42°C for 48 h followed by a 2 days recovery at 25°C. After this treatment, the survival rates of *CaWRKY27-OE4* and *OE9* seedlings were much lower than the WT seedlings (Supplementary Figure S2B). Second, traits related to thermosensitivity were assessed in 1-month-old *CaWRKY27*-*OE4*, *OE9*, and WT plants that were treated at 42°C for 48 h followed by a 48-h recovery at 25°C. HS-induced necrosis was clearly visible on *CaWRKY27*-*OE4* and *OE9* plants, while only minor necrosis was observed on WT plants (Supplementary Figure S2C). The same HS treatment and recovery was performed with 50-day-old WT, *CaWRKY27-OE4*, and *OE9* plants, the survival rates of *CaWRKY27-OE4* and *OE9* seedlings were much lower than the WT seedlings (Supplementary Figure S2D). We also tested the seed germination of *CaWRKY27-OE4*, *OE9*, and WT seeds by exposing them to 42°C for 15 h, and then returning them to 25°C to germinate. No significant difference in the percent seed germination was observed between *CaWRKY27-OE4*, *OE9*, and WT seeds at 25°C; however, significant differences were observed between *CaWRKY27-OE4* and *OE9* plants (26% and 25%, respectively) compared with WT plants (72%) at 8 days after treatment (DAT), as well as at 10 DAT (59% and 58% for *CaWRKY27-OE4* and *OE9*, respectively, and 81% for WT seeds) (Supplementary Figure S2E).

### The Expression of Heat Stress Defense Genes Is Induced in Plants Overexpressing *CaWRKY27*

**FIGURE 5:**
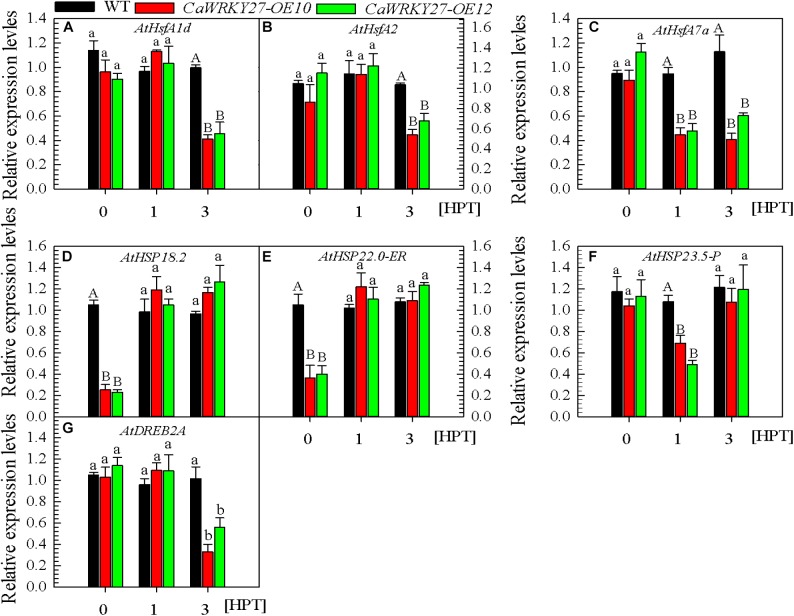
Analysis of thermotolerance-responsive gene expression levels by qRT-PCR in *CaWRKY27-OE10*, *OE12*, and WT plants at 1 and 3 h post treatment with heat stress. **(A–C)** The relative transcript levels of heat stress response genes *AtHsfA1d, AtHsfA2*, and *AtHsfA7a.* **(D–F)** The relative transcript levels of *HSP* genes *AtHSP18.2*, *AtHSP22.0-ER* and *AtHSP23.5-P*. **(G)** The relative transcript levels of transcription factor *AtDREB2A.* The transcript levels of different gene were normalized against *AtUBQ10*, followed with normalization against the gene in WT at 0 hpt. Data represent the mean ± SE of three biological replicates. Different letters indicate significant difference compared to WT (*SNK*-test, lowercase difference *P* < 0.05 or uppercase difference *P* < 0.01).

To confirm the indicated role for *CaWRKY27* as a negative regulator of thermotolerance and test its possible mode of action, we examined the effect of *CaWRKY27* overexpression on the transcript abundance of HS-response marker genes in transgenic tobacco and *Arabidopsis* under HS. We measured the ROS detoxification-associated genes *NtGST1* and *NtCAT1* (Takahashi et al., 1997), the ethylene biosynthesis-associated genes *NtACC deaminase*, *NtACS1*, *NtACS6*, *NtEFE26*, and *NtACC Oxidase* (Chen et al., 2003), and the thermotolerance-associated genes *NtHSF2*, *NtHSP18*, and *NtHSP90*. Our results showed that, in plants treated at 42°C, transcript levels of these genes were lower at 24 and 48 h post treatment (hpt) in transgenic tobacco than in WT (Supplementary Figure S3). The transcript levels of thermotolerance-associated genes, including the TF genes *AtHsfA1d*, *AtHsfA2* (Nishizawa-Yokoi et al., 2011), *AtHsfA7a*, and *AtDREB2A* (Sakuma et al., 2006), and the chaperone genes *AtHSP18.2*, *AtHSP22.0-ER*, and *AtHSP23.5-P* (Kotak et al., 2007; Ohama et al., 2016) were also measured by qRT-PCR in HS challenged or unchallenged plants. *AtHSP18.2* and *AtHSP22.0-ER* (Figures 5D,E) transcript abundances were significantly lower in *CaWRKY27-OE* lines under normal conditions compared with the WT. However, *AtHsfA1d*, *AtHsfA2*, *AtHsfA7a*, *AtDREB2A*, and *AtHSP23.5-P* (Figures 5A–C,F,G) did not exhibit any difference in their transcript abundances compared to that in the WT plants. No significant differences were observed in the transcript abundance of *AtHsfA1d*, *AtHsfA2*, and *AtDREB2A* between WT and *CaWRKY27-OE* plants at 1 hpt, yet the transcript abundance of these genes, as well as *AtHsfA7a* (Figure 5C), decreased significantly in *CaWRKY27-OE* lines at 3 hpt, compared with WT plants. Additionally, the expression of *AtHSP23.5-P* was significantly repressed in *CaWRKY27-OE* lines at 1 hpt, compared to WT plants (Figure 5F). Together, these data indicate that the overexpression of *CaWRKY27* negatively regulated basal thermotolerance in *Arabidopsis* plants by modulating the expression of HS response marker genes.

### H_2_O_2_ Accumulated in the Heat Stress Recovery Stage in Pepper

**FIGURE 6:**
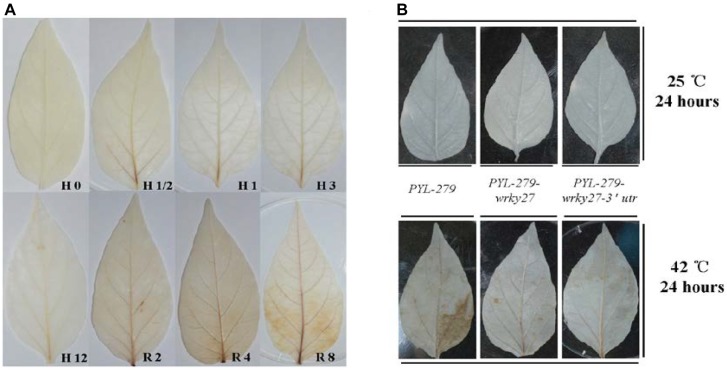
H_2_O_2_ accumulated in response to heat treatment. **(A)** Accumulation of H_2_O_2_ was detected in pepper leaves by DAB staining after 0, 0.5, 1, 3, and 12 h at 37°C and after 2, 4, and 8 h of recovery at 25°C. **(B)** The accumulation of H_2_O_2_ detected by DAB staining after 0 and 24 h of heat treatment in *PYL-279*, *PYL-279-wrky27*, and *PYL-279-wrky27-3*’*utr* pepper leaves.

H_2_O_2_ is the most stable ROS and acts as a signaling molecule in plant defense responses, including the pathogen response and HS response (Mittler, 2002; Mittler et al., 2004). We speculated that H_2_O_2_ might act as a signaling molecule upstream of *CaWRKY27*, since *CaWRKY27* regulates both pepper immunity against *R. solanacearum* (Dang et al., 2014) and thermotolerance. To test this hypothesis, we measured H_2_O_2_ accumulation and *CaWRKY27* transcript abundance in pepper plants during heat treatment, or during their recovery at room temperature. No significant increase in the abundance of H_2_O_2_ was observed by diaminobenzidine (DAB) staining at 0.5 to 12 hpt (37°C) in pepper leaves, but a significant increase in the abundance of H_2_O_2_ was observed after 2, 4, and 8 h recovery at 25°C in pepper leaves (Figure 6A). DAB staining also detected significant H_2_O_2_ accumulation in WT pepper leaves (*PYL-279*) that were challenged with 42°C for 24 h (Figure 6B), but not in WT and *CaWRKY27*-VIGS pepper leaves at 25°C. This shows that that H_2_O_2_ accumulation was triggered by high temperatures and its presence persisted into the recovery phase in pepper plants.

**FIGURE 7:**
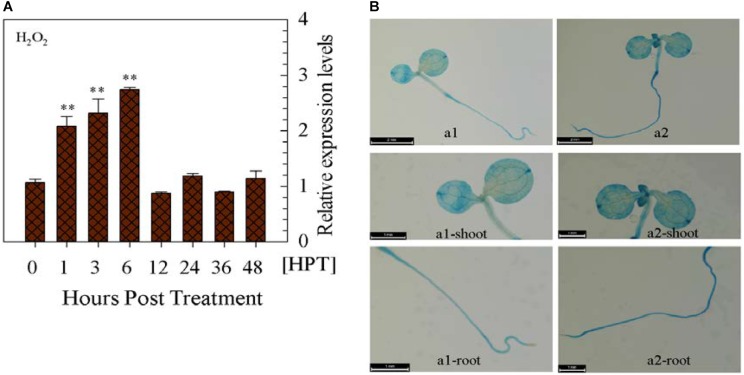
*CaWRKY27* gene expression was induced by exogenously applied H_2_O_2_ in pepper plants. **(A)** *CaWRKY27* expression in the leaves of WT pepper plants treated with H_2_O_2_ as determined by qRT-PCR. Relative *CaWRKY27* expression levels were compared between H_2_O_2_-treated pepper plants and mock-treated control plants, which were set to a relative expression level of ‘1.’ Data represent the mean ± SE of three biological replicates, and asterisks indicate significant differences compared to control plants (*SNK*-test, ^∗^*P* < 0.05 or *^∗∗^P* < 0.01). HPT, hours post treatment. **(B)** Seedlings carrying the *pCaWRKY27::GUS* construct were treated with H_2_O_2_ (10 mM) for 3 h. The a1-shoot, a1-root, a2-shoot, and a2-root, photographs are magnified images of the shoot or root of the corresponding a1 or a2 seedlings.

Upon the exogenous application of H_2_O_2_, *CaWRKY27* expression gradually increased over time from 1 to 6 hpt (Figure 7A). To further confirm this result, the expression of GUS driven by *pCaWRKY27* was enhanced at 3 h post treatment with exogenous H_2_O_2_ (Figure 7B). On contrast, *CaWRKY27* expression was significantly repressed in pepper isolated leaves when HS-induced ROS accumulation was cleared after 30 min recovery following heat treatment at 33°C for 3 h via 10 mM AsA, 100 μM DPI and 100 μM quinacrine (Supplementary Figures S4A,B). All these data indicate that *CaWRKY27* might act downstream of H_2_O_2_ in pepper HSR.

### Expression of ROS-Scavenging Enzymes and H_2_O_2_ Accumulation Were Affected by *CaWRKY27* Silencing in Pepper Plants

**FIGURE 8:**
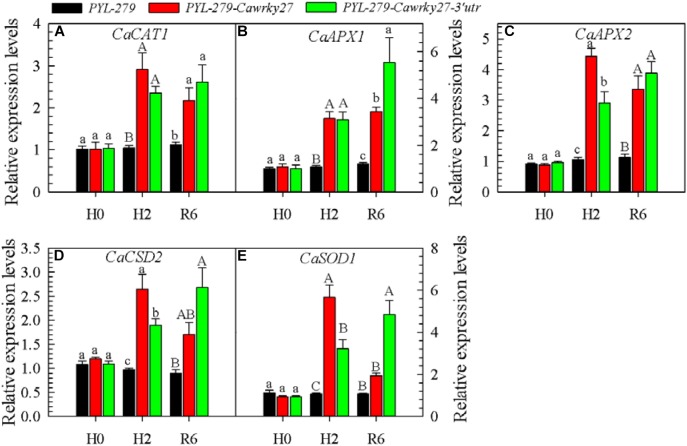
Analysis of defense-associated gene expression in *CaWRKY2*7-silenced plants. The silenced (*PYL-279-wrky27* and *PYL-279-wrky27-3’utr*) and control (*PYL-279*) pepper plant leaves were harvested after 0 and 2 h at 37°C, and after 6 h of recovery at 25°C (after 2 h of heat treatment). **(A–E)** Relative transcript levels of genes encoding the ROS-scavenging enzymes *CaCAT1*, **(A)**; *CaAPX1*, **(B)**; *CaAPX2*, **(C)**; *CaCSD2*, **(D)**; and *CaSOD1*, **(E)**. The transcript level of each gene was determined by qRT-PCR and normalized against *CaActin*, followed by normalization against the transcript level of the gene in *PYL-279.* Data represent the mean ± SE of three biological replicates. Different letters indicate significant differences compared to the control (*SNK-*test, lowercase letters indicate *P* < 0.05 and uppercase letters indicate *P* < 0.01).

To further confirm the relationship between CaWRKY27 and ROS accumulation, we investigated the expression of ROS-scavenging enzymes, including *CaCAT1*, *CaAPX1*, *CaAPX2*, *CaCSD2*, and *CaSOD1*, in *CaWRKY27*-silenced pepper plants. The results show that the expression levels of *CaCAT1*, *CaAPX1*, *CaAPX2*, *CaCSD2* and *CaSOD1* (Figures 8A–E) were significantly higher in *CaWRKY27*-silenced pepper plants than that in the WT exposed to 37°C for 2 h, and also at 6 hpt. No significant differences in the expression of these genes were detected under normal growth conditions (Figure 8). In accordance with this finding, H_2_O_2_ accumulation was significantly higher in the control plant (*PYL-279*) leaves than that in the *CaWRKY27*-silenced pepper leaves when the plants were challenged with 42°C for 24 h. This suggests that *CaWRKY27* silencing induces the expression of ROS-scavenging enzyme genes that in turn reduces the accumulation of ROS, including H_2_O_2_.

## Discussion

The HS caused by global climate change adversely affects plant growth and development, damaging crop yields and threatening food security. Transcriptome analyses identified a subset of WRKY TFs that were transcriptionally modulated in response to HS (Karanja et al., 2017; Zhang et al., 2017), implying that they might participate in the regulation of the plant HS response. However, information on the roles of WRKY TFs in regulating plant thermotolerance remains limited. We have previously shown that *CaWRKY27* acts as a positive regulator of the pepper response to *RSI* (Dang et al., 2014); in the present study we provide new evidence that *CaWRKY27* also acts as a negative regulator of basal thermotolerance in pepper, and that this regulation is mediated by H_2_O_2_ signaling.

Our qRT-PCR and GUS-promoter fusion analysis showed that *CaWRKY27* expression was induced by HS in *Arabidopsis*, suggesting that *CaWRKY27* might be involved in the HS response in pepper. This was further confirmed by VIGS-mediated silencing of *CaWRKY27* expression in pepper and ectopic overexpression of *CaWRKY27* in tobacco and *Arabidopsis*. The *CaWRKY27*-silenced pepper plants exhibited enhanced thermotolerance that manifested as reduced leaf wilting, water loss, and ion leakage, compared with the control plants. By contrast, the *Arabidopsis* plants overexpressing *CaWRKY27* exhibited decreased thermotolerance, as shown by their lower survival rates and seed germination ratio, as well as higher leaf ion leakage compared to the control plants. Similar phenotypes were also observed in tobacco plants that overexpress *CaWRKY27*. These results were further supported by our assessment of the expression of thermotolerance-associated genes, including *AtHsfA1d* (Nishizawa-Yokoi et al., 2011), *AtHsfA2* (Nover et al., 1996), *AtHsfA7a* (Larkindale and Vierling, 2008), *AtHSP18.2* (Liu et al., 2005), *AtHSP22.0-ER* (Wang et al., 2016), *AtHSP23.5-P* (Kirschner et al., 2000), and *AtDREB2A* (Reis et al., 2014) in *CaWRKY27*-overexpressing *Arabidopsis* plants. All of these genes displayed a decrease in expression during at least one tested time point during or after HS treatment compared to the control plants. Accordingly, thermotolerance-associated genes, including *NtHSP18* (Park et al., 2015), *NtHSP90* (Rizhsky et al., 2002), and *NtHSF2* (Shoji et al., 2000), decreased in expression in the *CaWRKY27*-overexpressing tobacco plants under heat treatment compared to WT plants.

Since the plant defense response to various stresses is generally resource intensive, activated at the expense of other biological processes, or highly deleterious to the host, defense responses need be tightly regulated to prevent inappropriate stress responses during stress, depress unnecessary defense in the absence of stress, or to inhibit the response after the stress has passed. Therefore, plants require both positive and negative regulators of their stress responses. Since *CaWRKY27* was induced during HS and remained elevated throughout the recovery from HS, *CaWRKY27* might act as a negative regulator of HS responses in pepper to block inappropriate HS responses, and importantly, to block these responses in pepper plants during their recovery from HS at room temperature. Similarly, a *NAC* TF (*SlJA2*) from tomato was also found to act as a negative regulator of basal thermotolerance (Liu et al., 2017).

Extensive crosstalk has been identified within or between plant responses to biotic and abiotic stresses and is believed to provide great regulatory potential that coordinates the various responses to different stresses (Fujita et al., 2006). Multiple studies suggest that a single WRKY TF might be involved in regulating several seemingly disparate processes (Rushton et al., 2010; Dang et al., 2013; Cai et al., 2015). The results from our present and previous studies show that *CaWRKY27* is upregulated by *RSI* and acts as a positive regulator of resistance to *RSI* in pepper (Dang et al., 2014). This suggests that *CaWRKY27* plays a role in the crosstalk between the pathogen and HS responses in pepper. So far, WRKY TFs from various plant species such as *CaWRKY6* (Cai et al., 2015), *CaWRKY40* (Dang et al., 2013), and *AtWRKY33* (Zheng et al., 2006; Li et al., 2011; Liu et al., 2015) have been found to act as a positive regulators of both plant immunity and thermotolerance. Unlike these WRKY TFs, *CaWRKY27* acts as a negative regulator of thermotolerance, but as a positive regulator of immunity against *R. solanacearum* in pepper. *CaWRKY27* upregulation upon pathogen attack might enable pepper to activate the immune response against infection by the pathogen, and to recruit more resources for immunity by blocking unnecessary HS responses.

In plants, ROS have been implicated as crucial signaling components in the crosstalk between the biotic and abiotic stress responses (Fujita et al., 2006). H_2_O_2_ is the most stable ROS and acts as a signaling molecule in defense responses, including responses to pathogens (Levine et al., 1994; Alvarez et al., 1998; Morales et al., 2016) and abiotic stresses such as heat (Dat et al., 1998). Accordingly, we observed significant H_2_O_2_ accumulation in WT pepper leaves at 42°C for 24 h, but no H_2_O_2_ accumulation was detected in WT pepper leaves or *CaWRKY27*-VIGS pepper plants under non-stressed conditions. However, HS rapidly enhanced intracellular production of H_2_O_2_, approximately 2.3-fold at 37°C and 2.5-fold at 42°C within a 1-h treatment in *Arabidopsis*, suggesting different HS response mechanisms between pepper and *Arabidopsis*. This difference may be due to the heightened temperature sensitivity in *Arabidopsis*, evident by its inability to survive prolonged HS, a trait that may be due to local adaptation of the genus (Volkov et al., 2006).

The data in the present study established a close relationship between the transcriptional expression of *CaWRKY27* with ROS accumulation during pepper’s HS response. ROS accumulation and transcriptional expression of *CaWRKY27* triggered by HS were significantly blocked by application of inhibitors of NADPH-oxidase, PA-Oxidase or ascorbic acid at 30 min of recover from HS, since NADPH-oxidase and PA-Oxidase are responsible for ROS accumulation during plant response to biotic or abiotic stress (Moller, 2001; Yoda et al., 2006; Andronis et al., 2014; Ben Rejeb et al., 2015; Karkonen and Kuchitsu, 2015; Gemes et al., 2016), and ascorbic acid is a ROS scavenger, it can be speculated that that both the ROS accumulation and transcription of *CaWRKY27* were conferred by ROS production or the inhibition of ROS degradation. More importantly, exogenous application of H_2_O_2_ significantly triggered the transcription of *CaWRKY27.* All these results placed H_2_O_2_ upstream *CaWRKY27* as a signaling components during pepper response to HS. Similarly, *HsfA1a* has been found to be regulated by H_2_O_2_ that accumulates in response to various stresses (Zhou et al., 2018). Given the existence of multiple HS sensors in plant cells (Mittler et al., 2012; Srivastava et al., 2014), the multiple H_2_O_2_ biosynthesis sites [apoplast and chloroplasts, mitochondria and peroxisome (Pellinen et al., 1999; Ozgur et al., 2015; Saxena et al., 2016)] and close relationship between H_2_O_2_ accumulation and Ca^2+^ signaling cascades (Larkindale and Huang, 2004; Qiao et al., 2015), phytohormones [SA, JA, ET, and ABA (Larkindale and Huang, 2004; Oh et al., 2005)] or MAPK cascade (Song et al., 2015), the transduction of H_2_O_2_ mediated HS signaling into the nuclei might be performed via complicated signaling networks. In addition, it is also possible that H_2_O_2_ might modulate TFs through direct oxidation, since it was recently found that the oxidation of BRASSINAZOLE-RESISTANT1 (BZR1) TF can be induced via H_2_O_2_, and played a major role in regulating gene expression (Tian et al., 2018). To elucidate the complicated molecular link between H_2_O_2_ accumulation and *CaWRKY27* transcription, further investigation is required.

Interestingly, leaves from *CaWRKY27*-overexpressing *Arabidopsis* plants exhibited more H_2_O_2_ accumulation than control plants, and no significant H_2_O_2_ accumulation was observed *CaWRKY27*-silenced pepper leaves under HS, reflecting a difference in H_2_O_2_ accumulation between the response of *Arabidopsis* and pepper to heat, which might be due to their evolution under different ecological conditions. The positive feedback regulation of H_2_O_2_ by *CaWRKY27* in pepper plants might be due to the *CaWRK27*-dependent derepression of ROS-scavenging enzyme genes, including *CaCAT1*, *CaAPX1*, *CaAPX2*, *CaCSD2*, and *CaSOD1*. Derepression was evident since the transcript levels of these genes were significantly lower in heat-challenged *CaWRKY27*-silenced pepper plants than in control plants. This result is consistent with our previous study that found a higher level of H_2_O_2_ accumulation in *CaWRKY27*-overexpressing tobacco plants challenged with *RSI* (Dang et al., 2014). Similarly, overexpression of tomato *SlJA2*, a negative regulator of the HS response, in tobacco also represses expression of ROS-scavenging genes (Liu et al., 2017). The positive feedback between H_2_O_2_ accumulation and *CaWRKY27* expression further supports the speculation that H_2_O_2_ might act as a signaling component upstream of C*aWRKY27*.

Some of the thermotolerance-associated marker genes such as *AtHsfA1d*, *AtHsfA2*, *AtHsfA7a*, *AtDREB2A*, and *AtHSP23.5-P* exhibited different expression in unchallenged and heat-treated plants at 1 and 3 hpt. More interestingly, *CaCAT1*, which was activated by overexpression of *CaWRKY27* in transgenic tobacco plants that were inoculated with *R. solanacearum* (Dang et al., 2014), was downregulated by overexpression of *CaWRKY27* in heat-treated transgenic tobacco plants in the present study. One possible explanation for these contradictory results might be that *CaWRKY27* function may be modulated by protein–protein interactions that are governed by other signaling inputs that are activated by different stresses. In support of this, some WRKY TFs were found to be functionally modulated by physically interacting with a wide range of proteins with roles in signaling, transcription, and chromatin remodeling (Chi et al., 2013; Alves et al., 2014; Tripathi et al., 2015). Further isolation and functional characterization of potential protein interactors of CaWRKY27 in pepper plants challenged with various stimuli will provide insight into the role of *CaWRKY27* in various stress responses.

Collectively, the data in the present study, together with those of our previous study, suggest that *CaWRKY27* is a positive regulator of the *RSI* response and a negative regulator of the HS response in pepper. *CaWRKY27*-dependent regulation of the HS response is mediated by H_2_O_2_-associated signaling, and blocks unnecessary responses during the *RSI* response, during recovery from HS, and prevents an inappropriate response during HS challenge in pepper.

## Author Contributions

SH and YW designed the experiments. FD and JL performed most of experiments and analyzed the data. The other authors assisted in experiments and discussed the results. FD and SH wrote the manuscript. All authors read and approved the manuscript.

## Conflict of Interest Statement

The authors declare that the research was conducted in the absence of any commercial or financial relationships that could be construed as a potential conflict of interest.

## Supplementary Material

FIGURE S1 *CaWRKY27* promoter motifs and expression in silenced and overexpression lines. **(A)** Nucleotide sequences from the *CaWRKY27* 5’ flanking promoter region. The four heat stress response elements (*HSEs*) that may act as *cis*/*trans* motifs are marked by arrows. **(B)** Relative expression of *CaWRKY27* in *PYL-279*, *PYL-279-wrky27*, and *PYL-279-wrky27-3*’*utr* pepper plants that had been either unchallenged or challenged with heat stress. *CaWRKY27* relative expression was normalized against *CaActin*, followed by normalization against the *CaWRKY27* expression in the *PYL-279* control. Data represent the mean ± SE of three biological replicates. **(C)** Transcription of *CaWRKY27* in *CaWRKY27*-overexpressing *Arabidopsis* plant lines was determined via semi-quantitative PCR, and normalized against *AtUBQ10* expression.

FIGURE S2 Phenotypes of *CaWRKY27*-overexpressing tobacco lines. **(A)** Relative expression of *CaWRKY27* was analyzed in nine *CaWRKY27*-overexpressing lines and WT (K326) plants with qRT-PCR. *CaWRKY27* expression was normalized against *NtEF1α*, followed by normalization against the *CaWRKY27* expression in the WT. Data represent the mean ± SE of three biological replicates. **(B–D)** Phenotypes of 15-, 30-, and 55-day-old *CaWRKY27*-overexpressing lines and WT plants that were treated at 42°C for 48 h, then returned to 25°C to recover for 48 h. **(E)** Effect of heat on seed germination rate (percent of radicle emergence) was recorded daily until no further germination occurred. Seeds of WT, *CaWRKY27-OE4*, and *OE9* lines were treated at 42°C for 15 h and then returned to 25°C for germination. Data represent the mean (*n* = 8 at 25°C, *n* = 5 at 42°C) ± SE, and each replicate consisted of 32-34 seeds.

FIGURE S3 Expression of thermotolerance-associated genes were monitored by qRT-PCR in wild type (K326) and *CaWRKY27-OE4* plants at 24 and 48 h after heat stress (42°C). **(A,B)**, Relative expression of the ROS-scavenging enzyme genes *NtGST1* and *NtCAT1* in heat-treated *CaWRKY27-OE4* and WT plants. **(C–G)** Expression of the ethylene biosynthesis associated genes *NtACC deaminase*, *NtACS1*, *NtACS6*, *NtEFE26*, and *NtACC Oxidase*, in heat-treated *CaWRKY27-OE4* and WT plants. **(H)** Expression of the heat-shock factor *NtHSF2* in heat-treated *CaWRKY27-OE4* and WT plants. **(I,J)** Expression of the heat-shock proteins *NtHSP18* and *NtHSP90* in heat-treated *CaWRKY27-OE4* and WT plants. The transcript level of each gene was normalized against *CaActin*, followed by normalization against the transcript level of the gene in heat-treated WT plants. Data represent the mean ± SE of three biological replicates. Asterisks indicate significant differences compared with WT plants (*SNK*-test, ^∗^*P* < 0.05 or ^∗∗^*P* < 0.01).

FIGURE S4 *CaWRKY27* expression was repressed in isolated pepper leaves under heat stress with ROS scavenging reagent. **(A)** Accumulation of H_2_O_2_ were detected via DAB staining at 3 h after heat stress (33°C) and 30 min after recovery (25°C) with or without 10 mm AsA (ascorbic acid), 100 μM DPI (diphenyleneiodonium chloride) and 100 μM quinacrine in isolated pepper leaves. **(B)** *CaWRKY27* expression was determined via qRT-PCR at 3 h after heat stress (33°C) and 30 min after recovery (25°C) with or without 10 mm AsA (ascorbic acid) in isolated pepper leaves. Data represent the mean ± SE of three biological replicates.

TABLE S1 Sequences of primers used in this study.
