# Supplementary figures and images for: CaWRKY27 Negatively Regulates H2O2-Mediated Thermotolerance in Pepper (Capsicum annuum)
Source: Front Plant Sci. 2018 Nov 19;9:1633. doi: 10.3389/fpls.2018.01633 (PMC6252359; doi:10.3389/fpls.2018.01633)

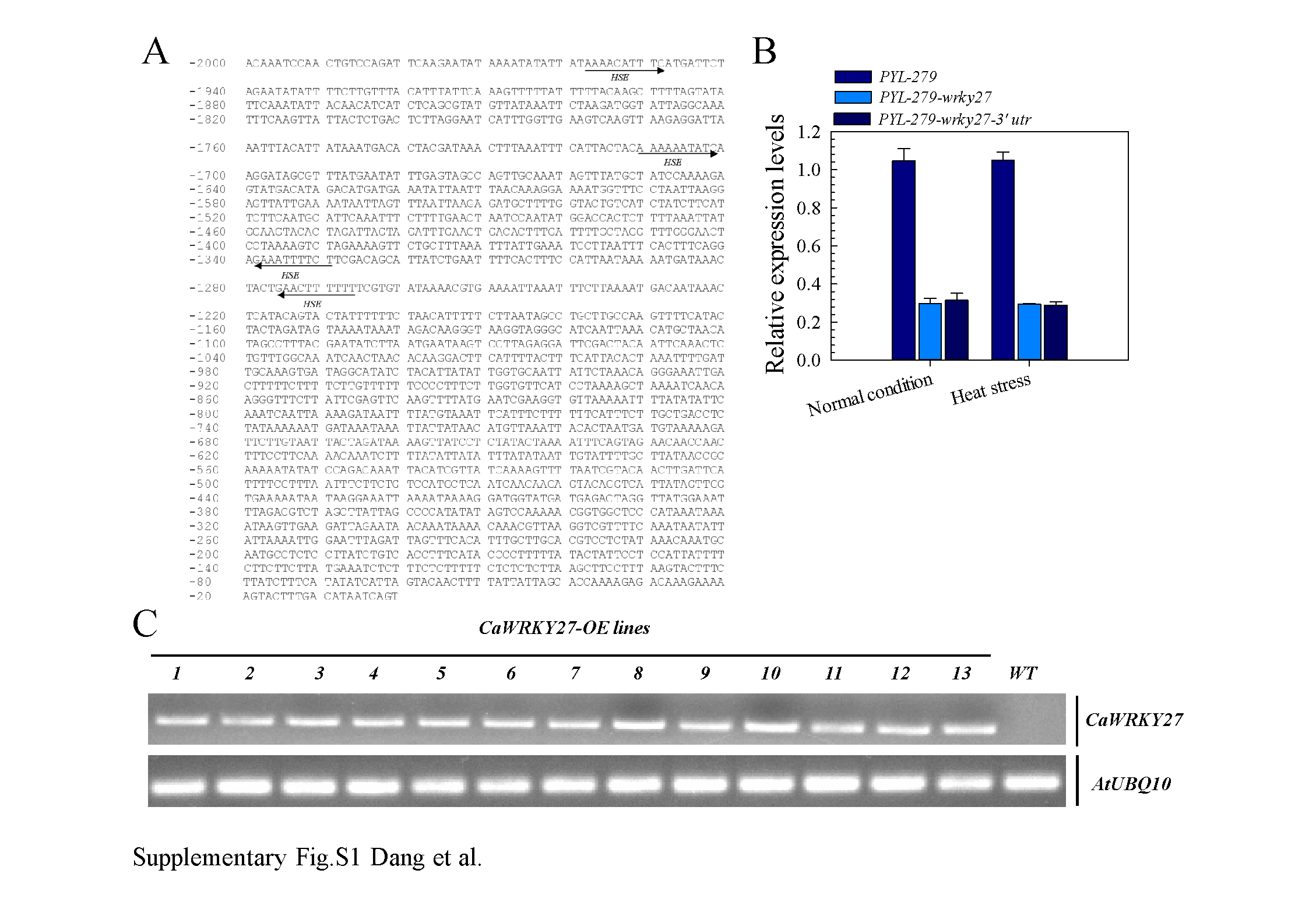

Supplement: FIGURE S1 — CaWRKY27 promoter motifs and expression in silenced and overexpression lines. (A) Nucleotide sequences from the CaWRKY27 5’ flanking promoter region. The four heat stress response elements (HSEs) that may act as cis/trans motifs are marked by arrows. (B) Relative expression of CaWRKY27 in PYL-279, PYL-279-wrky27, and PYL-279-wrky27-3’utr pepper plants that had been either unchallenged or challenged with heat stress. CaWRKY27 relative expression was normalized against CaActin, followed by normalization against the CaWRKY27 expression in the PYL-279 control. Data represent the mean ± SE of three biological replicates. (C) Transcription of CaWRKY27 in CaWRKY27-overexpressing Arabidopsis plant lines was determined via semi-quantitative PCR, and normalized against AtUBQ10 expression. [file Image_1.TIF]

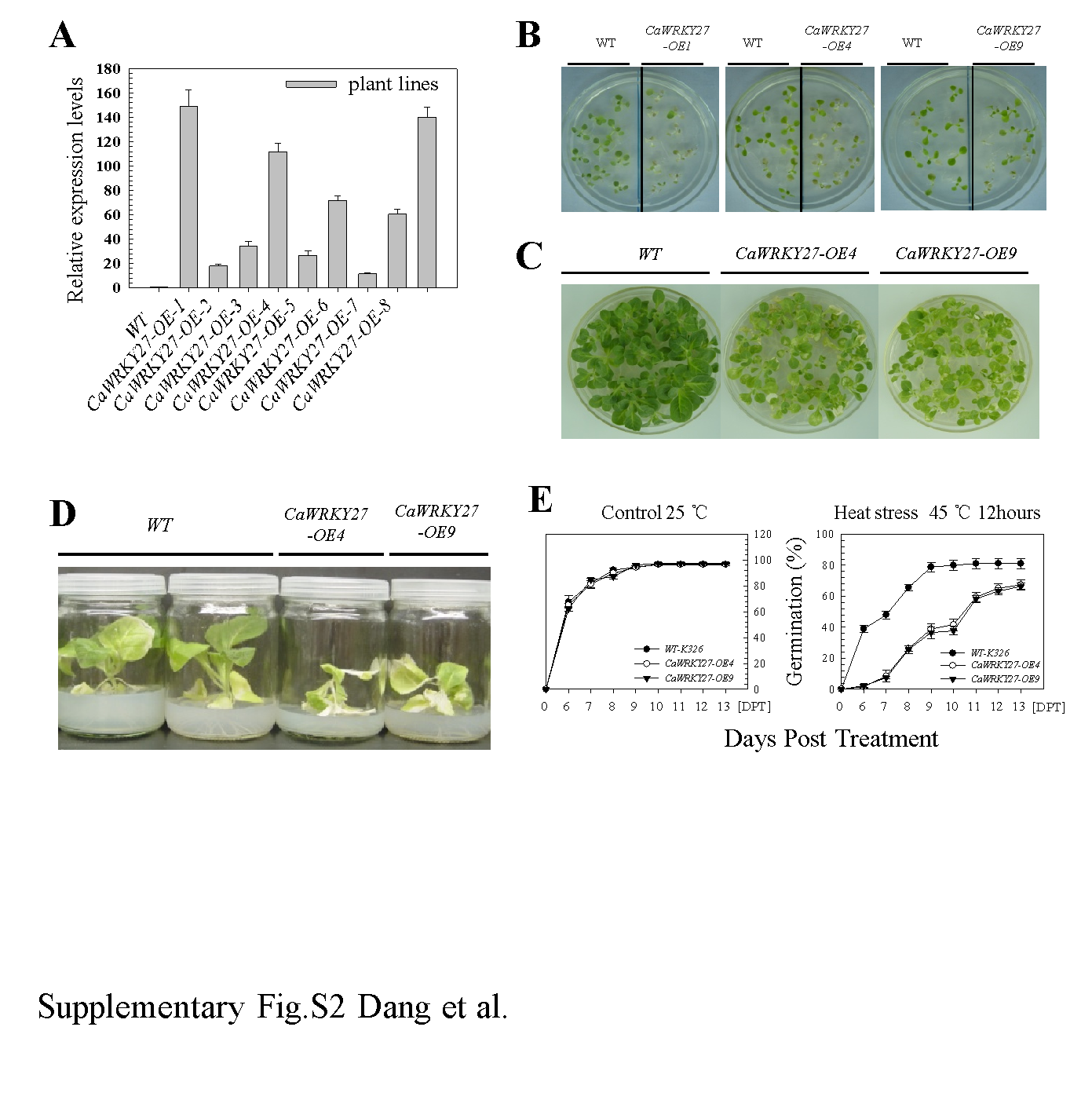

Supplement: FIGURE S2 — Phenotypes of CaWRKY27-overexpressing tobacco lines. (A) Relative expression of CaWRKY27 was analyzed in nine CaWRKY27-overexpressing lines and WT (K326) plants with qRT-PCR. CaWRKY27 expression was normalized against NtEF1α, followed by normalization against the CaWRKY27 expression in the WT. Data represent the mean ± SE of three biological replicates. (B–D) Phenotypes of 15-, 30-, and 55-day-old CaWRKY27-overexpressing lines and WT plants that were treated at 42°C for 48 h, then returned to 25°C to recover for 48 h. (E) Effect of heat on seed germination rate (percent of radicle emergence) was recorded daily until no further germination occurred. Seeds of WT, CaWRKY27-OE4, and OE9 lines were treated at 42°C for 15 h and then returned to 25°C for germination. Data represent the mean (n = 8 at 25°C, n = 5 at 42°C) ± SE, and each replicate consisted of 32-34 seeds. [file Image_2.TIF]

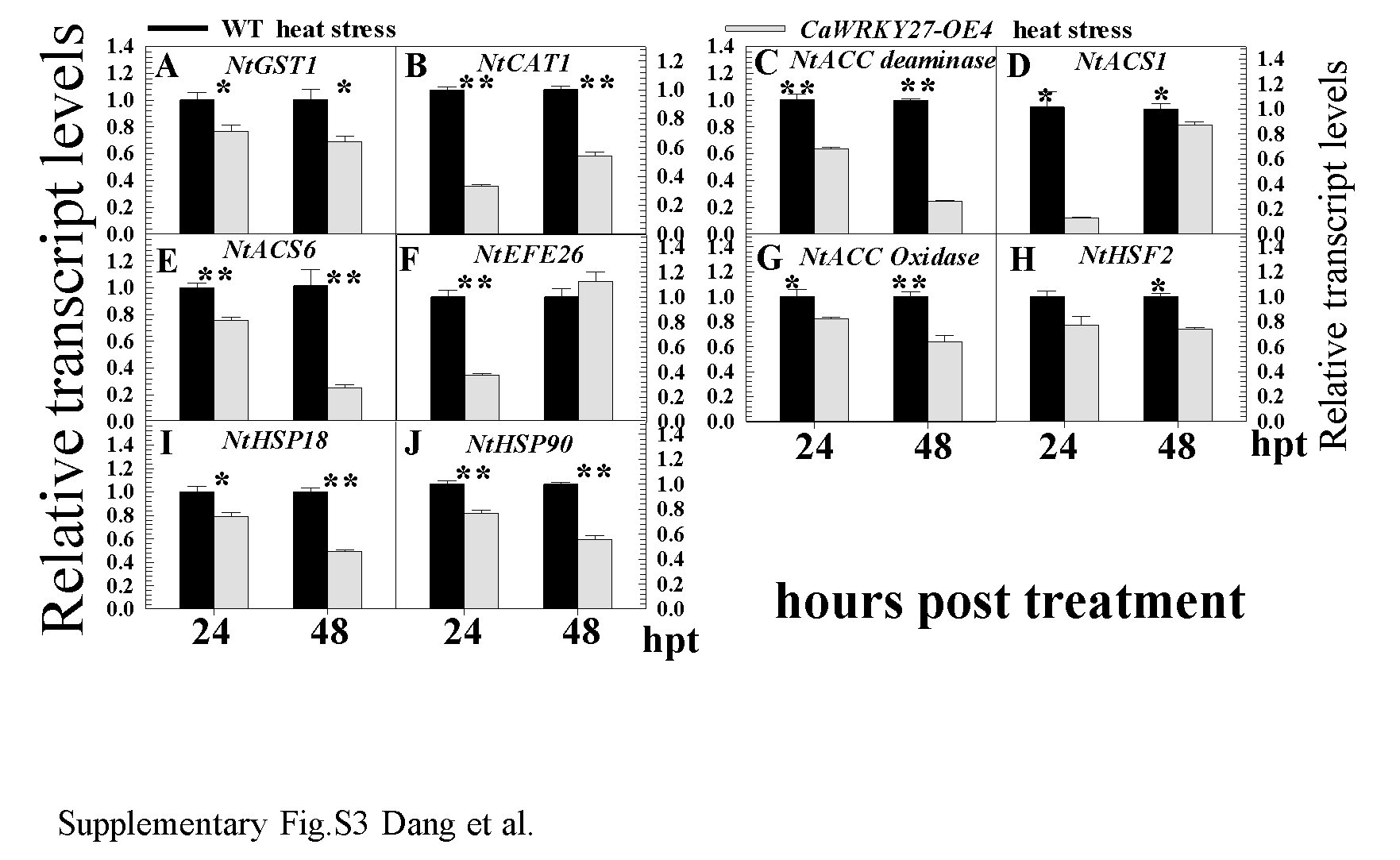

Supplement: FIGURE S3 — Expression of thermotolerance-associated genes were monitored by qRT-PCR in wild type (K326) and CaWRKY27-OE4 plants at 24 and 48 h after heat stress (42°C). (A,B), Relative expression of the ROS-scavenging enzyme genes NtGST1 and NtCAT1 in heat-treated CaWRKY27-OE4 and WT plants. (C–G) Expression of the ethylene biosynthesis associated genes NtACC deaminase, NtACS1, NtACS6, NtEFE26, and NtACC Oxidase, in heat-treated CaWRKY27-OE4 and WT plants. (H) Expression of the heat-shock factor NtHSF2 in heat-treated CaWRKY27-OE4 and WT plants. (I,J) Expression of the heat-shock proteins NtHSP18 and NtHSP90 in heat-treated CaWRKY27-OE4 and WT plants. The transcript level of each gene was normalized against CaActin, followed by normalization against the transcript level of the gene in heat-treated WT plants. Data represent the mean ± SE of three biological replicates. Asterisks indicate significant differences compared with WT plants (SNK-test, ∗P < 0.05 or ∗∗P < 0.01). [file Image_3.TIF]

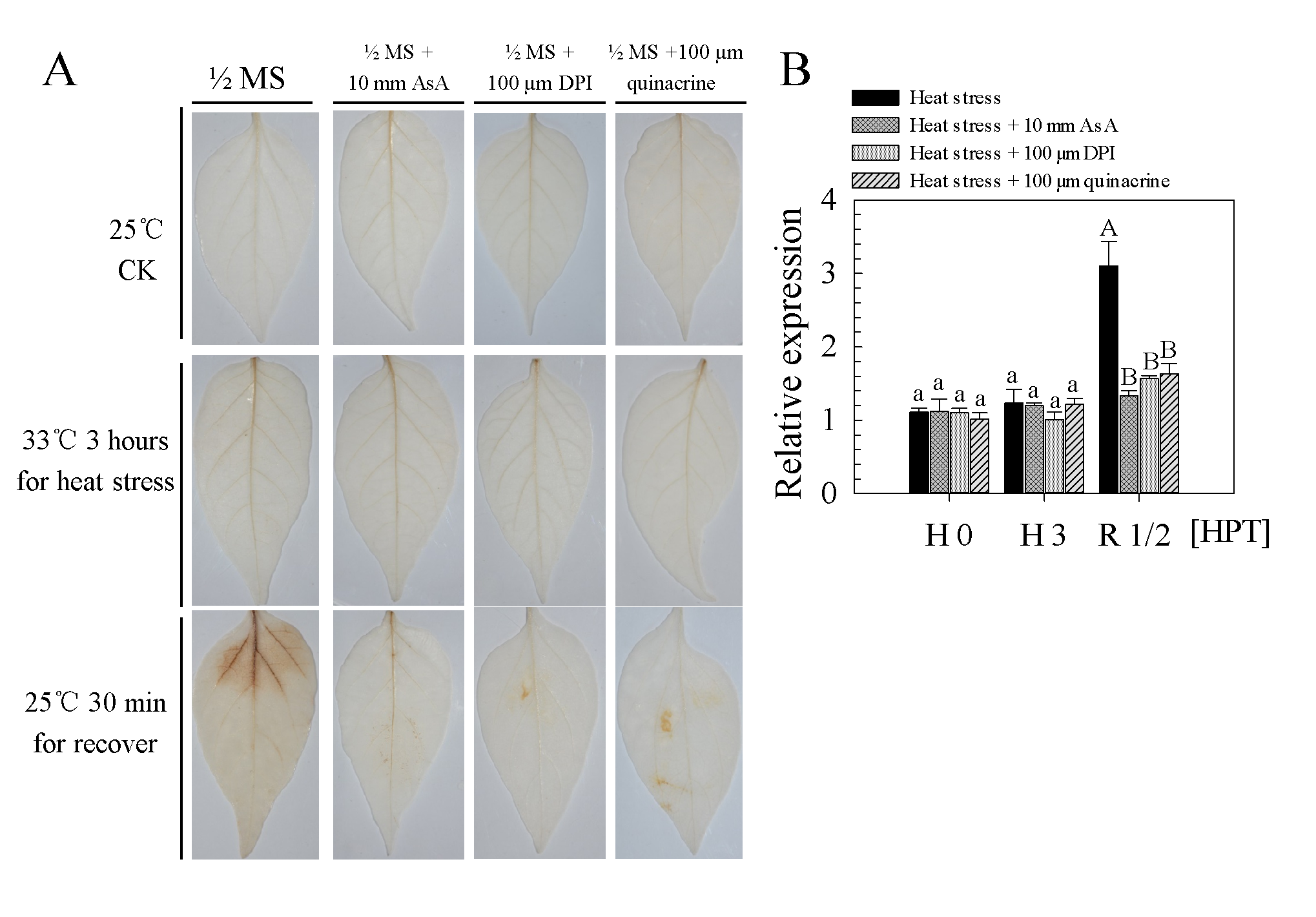

Supplement: FIGURE S4 — CaWRKY27 expression was repressed in isolated pepper leaves under heat stress with ROS scavenging reagent. (A) Accumulation of H2O2 were detected via DAB staining at 3 h after heat stress (33°C) and 30 min after recovery (25°C) with or without 10 mm AsA (ascorbic acid), 100 μM DPI (diphenyleneiodonium chloride) and 100 μM quinacrine in isolated pepper leaves. (B) CaWRKY27 expression was determined via qRT-PCR at 3 h after heat stress (33°C) and 30 min after recovery (25°C) with or without 10 mm AsA (ascorbic acid) in isolated pepper leaves. Data represent the mean ± SE of three biological replicates. [file Image_4.TIF]
