# Supplementary material for: CaWRKY27 Negatively Regulates H2O2-Mediated Thermotolerance in Pepper (Capsicum annuum)
Source: Front Plant Sci. 2018 Nov 19;9:1633. doi: 10.3389/fpls.2018.01633 (PMC6252359; doi:10.3389/fpls.2018.01633)
Supplement: TABLE S1 — Sequences of primers used in this study. [file Table_1.DOC]

**Table S1**. Sequences of primers used in this study

| Primer Name | Primer Sequences(5’-3’) |
| --- | --- |
| **For cloning*** | |
| *CaWRKY27-CDSF* | GGGGACAAGTTTGTACAAAAAAGCAGGCTTCATGGAAGGAAGATTCAACAA |
| *CaWRKY27-CDSR* | GGGGACCACTTTGTACAAGAAAGCTGGGTCTTAGCCTGTGGTCCCACACCAC |
| *CaWRKY27-PF* | GGGGACAAGTTTGTACAAAAAAGCAGGCTACAAATCCAACTGTCCAGAT |
| *CaWRKY27-PR* | GGGGACCACTTTGTACAAGAAAGCTGGGTACTGATTATGTCAAAGTACTT |
| *wrky27-VIGS-F* | GGGGACAAGTTTGTACAAAAAAGCAGGCTTCCGACTTCTGATATTCCGGTT |
| *wrky27-VIGS-R* | GGGGACCACTTTGTACAAGAAAGCTGGGTCCCCTTGAACATTCCGGTAACT |
| *wrky27-3’utr-VIGS-F* | GGGGACAAGTTTGTACAAAAAAGCAGGCTTCTATTTGGCCGTGGAATGATGG |
| *wrky27-3’utr-VIGS-R* | GGGGACCACTTTGTACAAGAAAGCTGGGTCTTATTTTACTATACTACAAAAC |
| **For qRT-PCR** | |
| *AtHsfA1d* | Forward: CGAGAGTAATAGGCGCATCAG  Reverse: CATTGCTTTGGCTTGCTCGT |
| *AtHsfA2* | Forward: TGGGATTCTCATAAGTTCTCAACA  Reverse: TGGATCAATCTTTCTGAATCCAT |
| *AtHsfA7a* | Forward: TGCATTCTTTCTCCACGATTC  Reverse: TCTGCTTCTATCTTTCTGAAACC |
| *AtDREB2A* | Forward: CAGTGTTGCCAACGGTTCAT  Reverse: AAACGGAGGTATTCCGTAGTTGAG |
| *AtHSP18.2* | Forward: TTACCGGAGAATGCAAAGATG  Reverse: CGGAGATATCGATGGACTTGA |
| *AtHSP23.5-P* | Forward: GATCAAGATGCGTTTCGACAT  Reverse: TTCTACAGAGATTTTGACGTCTTCTT |
| *AtHSP22.0-ER* | Forward: ACTACTCCAGGCAGCTTGCTA  Reverse: CTTGAATGGATCAGGGAACC |
| *AtUBQ10* | Forward: GGCCTTGTATAATCCCTGATGAATAAG  Reverse: AAAGAGATAACAGGAACGGAAACATAGT |
| *NtGST1* | Forward: AGCACCCTTACCTTTCCCTC  Reverse: GCTTTCCTTCACAGCAGCATCA |
| *NtCAT1* | Forward: CAACTTCCTGCTAATGCTCCAA  Reverse: TGCCTGTCTGGTGTGAATGA |
| *NtACC deaminase* | Forward: TCTGAGGTTACTGATTTGGATTGG  Reverse: TGGACATGGTGGATAGTTGCT |
| *NtACS1* | Forward: CATTAGCGAGGATTCGGAGTT  Reverse: GTGGTGAATGAGGGATAGGAGA |
| *NtACS6* | Forward: ATGCCAAGGAAAGGGATTCTACA  Reverse: TGGGAGGTTTGGGCGAAGA |
| *NtEFE26* | Forward: CGGACGCTGGTGGCATAAT  Reverse: CAACAAGAGCTGGTGCTGGATA |
| *NtACC Oxidase* | Forward: GACAAAGGGACATTACAAGAAGT  Reverse: GAGAAGGATT ATGCCACCAG |
| *NtHSF2* | Forward: GCACAGGCTTGTAAATTGGTGGAAA  Reverse: AGCAGAGCATCTAATGAGAGCAGAA |
| *NtHSP18* | Forward: AGAAACCCCAGATTCCCATA  Reverse: GGCAGCCTAA ACCTTCTCAT |
| *NtHSP90* | Forward: AGGTGGTTGTCTCTGACCGTGT  Reverse: GGAGGGCAGTCTCAAACAGCAA |
| *NtEF1α* | Forward: TGCTGCTGTAACAAGATGGATGC  Reverse: GAGATGGGGA CAAAGGGGATT |
| *CaWRKY27* | Forward: CTGAGCAAGATGATTCCGAGAA  Reverse: ATTGGCACTGACACCACTCT |
| *CaCAT1* | Forward: TGTTGCTGGTGTTGGTGTTGGT  Reverse: GCCTCTCCTAGACGGCCTTTCA |
| *CaAPX1* | Forward: CAGGCAGAGGCAAGGGAGGTAA  Reverse: CCCTACGAGCGAGCCTACGAAT |
| *CaAPX2* | Forward: TGATGCTCTGCCAATATCGGAT  Reverse: CCAATTCCTCCCATGCCAACA |
| *CaCSD2* | Forward: TGTGGATGTGGCTTTGGTTCCC  Reverse: TGAGGAAGGGAGTTTGCGACAC |
| *CaSOD1* | Forward: CGATGGGAGGTTGAGCAATGGA  Reverse: TCCTGGCTTCTTGTTCGGTTGA |
| *CaPIN2* | Forward: CAATCCCAAGCCTCCCAAGTCT  Reverse: TGGGTCAGATTGTCCTTCGCAA |
| *CaACC Oxidase* | Forward: GGGACTCCGTGCTCACACAGAT  Reverse: TCGCCAAGGTTGACCACAATGG |
| *CaActin* | Forward: AGGGATGGGTCAAAAGGATGC  Reverse: GAGACAACACCGCCTGAATAGC |

*Sequences of the recombination sites of the Gateway system were underlined in these primers
